# Mesoporous silica nanoparticles prepared by different methods for biomedical applications: Comparative study

**DOI:** 10.1049/nbt2.12023

**Published:** 2021-02-22

**Authors:** Mohamed M. Ashour, Mostafa Mabrouk, Islam E. Soliman, Hanan H. Beherei, Khairy M. Tohamy

**Affiliations:** ^1^ Faculty of Engineering Badr University in Cairo (BUC) Cairo Egypt; ^2^ Refractories, Ceramics and Building Materials Department National Research Centre Giza Egypt; ^3^ Biophysics Branch Faculty of Science Al‐Azhar University Cairo Egypt

## Abstract

In the current investigation, mesoporous silica nanoparticles were obtained by various techniques, namely sol–gel (S1), micro‐emulsion (S2) and hydrothermal synthesis (S3). The effect of those methods on the final features of the obtained mesoporous silica nanoparticles was studied. The obtained nanoparticles were investigated by TEM, BET surface area, Zetasizer, XRD and FTIR. The preparation method effect was evaluated on the drug release behaviour using doxycycline hyclate as a model drug. In addition, the degree of their compatibility against Saos‐2 cell line was also determined. The morphology and microstructure of silica nanoparticles were found to be dependent on the utilised method. Those techniques produced particles with particle sizes of 50, 30–20 and 15 nm and also surface areas of 111.04, 164 and 538.72 m^2^/g, respectively, for S1, S2 and S3. However, different preparation methods showed no remarkable changes for the physical and chemical integrities. The drug release test showed faster release from S2 compared with S1 and S3, which make them more applicable in cases require large doses for short periods. However, the release behaviour of S3 was satisfied for treatments which require long period with relatively highest release rate. The preparation method influenced the cell viability as S1 and S2 showed acceptable cell cytotoxicity compared with S3.

## 1 INTRODUCTION

Drug delivery is the administration of therapeutic compounds to improve specific therapeutic issues within the human body. The technologies related to drug delivery are considerably developed to enhance the therapeutic properties, immunity response, nature of delivered materials [1], efficiency and safety of the drug to be suitable for certain diseases treatment [2] and avoiding short falls as low bioavailability [3], as well as drug decomposition. Due to desired therapeutic characteristics of the delivery system, such as delivering drug to the target tissues in human body without side effects to the other parts of body, a wide area of research that aims to develop the carrier properties to be more applicable for this application has been on the rise.

For instance, to avoid acute side effects as toxicity, the concentration of drug should be controlled. Therefore, the optimum concentration of drug could be maintained by controlling the release rate of drug. To achieve the desired aims, various materials have been extensively tested such as liposomes [4], dendrimers [5, 6], amphiphilic block copolymers [7, 8], hydrogels [9, 10] and inorganic‐based nanoparticles [2, 11, 12]. Among numerous drug delivery systems, inorganic‐based nanoparticles have demonstrated extraordinary physical and chemical properties, which make them magnificent drug carriers. As one of the inorganic carriers, the mesoporous silica nanoparticles (MSNs) which contain the features to achieve all the above aimed functions, is considered a promising candidate for those applications.

MSNs have been through several stages since their discovery, firstly, Stober et al. [13] have prepared silica particles of 1 µm size. Moreover, silica‐based nanomaterials with scales that vary from few hundred nanometres to several micrometres were produced using controlled hydrolysis of TEOS as a silica precursor [14]. Preparing hexagonal arrayed mesoporous structured silica nanoparticles have been synthesised by using binary surfactant method [15, 16]. They were employed with triethanolamine as the base and cationic surfactant as the template to prepare nanosized MSNs. The shape and size of the manufactured nano‐silica‐based drug delivery system could be controlled with the addition of electrolytes, organic acids or surfactants. Utilising this knowledge, Kim et al. [17], have synthesised silica nanoparticles using sodium iodide electrolyte. Rahman et al. [18] had also used ammonium bromide to control the size of produced nanoparticles. Furthermore, MSNs were prepared with the use of (CTAB) cetyltrimethyl ammonium bromide and sulphonated aromatic poly(ether ether ketone) (SPEEK) or poly(allyl amine hydrochloride) (PAACl) as a co‐template under basic conditions by Pang et al. [19]. Also Wang et al. [20], had employed tartaric acid to prepare cubic silica nanoparticles.

With unique physical characteristics and morphological structure, the two‐dimensional (2D) hexagonal uniformed size particles MSNs in the range of 80–500 nm, high specific surface areas of about below 1000 m^2^/g, large volume of pores in the range of 0.5–2.5 cm^3^/g and the narrow range of diameters of pores was recognized (1.3–30 nm). It was earlier highlighted that particles possess controllable morphology of both interior and exterior surfaces, which are very suitable for several surface modifications and are extensively utilised for drug delivering applications [21]. It is also well known that water addition after the hydrolysis of tetraethoxysilane would produce silica nanopowders with monodispersed particles pf size of about 10.6 nm as previously reported [22]. Burglova et al. [23], had used MSNs as a carrier for encapsulation of oncological drug 3‐hydroxyquinolinone (3‐HQ). They studied the effect of CTAB and TEOS content on the characteristics of synthesised nanoparticles. They found that as a result of increasing the concentration of TEOS twice at a constant concentration of CTAB, the particle size would increase. [TEOS]:[CTAB] ratio of 8, 4 and 2 considerably produced particle sizes of 177, 510 and 1141 nm. Stanley et al. [24] checked the effect of the nature of the surfactant on the size of the prepared nanoparticles. They had amorphous silica nanoparticles with average particle size that varied from 296.7 to 608.6 nm by hydrolysis of TEOS in ethanol with addition of different concentrations of CTAB, PVP and SDS as surfactants.

In addition to, Venkatathri [25] found that spherical silica nanoparticles synthesised by homogeneous system of NH_4_OH, H_2_O, ethanol and TEOS had bimodal particle sizes with 80% of particles in the 100 nm range and 20% in the 300 nm range . He observed that the involvement of octyldecyltrimethoxy silane (ODTS) in the colloidal solution containing silica nano‐spheres could be changed to amorphous core/mesoporous shell with and uniform particle size of 400 nm and surface area of 93.54 (m^2^/g). Xu et al. [26], had dissolved decane into solution of CTAB and NH_4_OH with TEOS as a precursor to produce hollow mesoporous silica spheres with a diameterof about 650 nm, pore size of 2.48 nm and specific area of 1070 (m^2^/g). From all the previous examples, for MSNs which have been recorded in the last three decades, it can be suggested that MSNs could be obtained in different morphologies and porous structures using the same starting materials and pore formers. These variations in the MSNs important features create diversity in their application as medication delivery vehicles, especially in their drug delivery performances and mechanisms. It is also thought that these variations originated from the use of different preparation methods and techniques.

On considering the above factors,controlling the particle size and distribution is found to be dependent on the processing conditions of the MSNs. Therefore, our aim in this research work is to study the effect of different preparation methods of MSNs based on their final properties thereby making them a valued drug delivery system with perfect performance. For this purpose, a comparison study of the effect of the preparation method on the morphology, physicochemical properties, drug release behaviour and cytotoxicity of these MSNs as drug delivery systems is the focus of this article.

## 2 MATERIALS AND METHODS

### 2.1 Materials

Tetra ethyl orthosilicate (TEOS) SiC_8_H_20_O_4_ with a molecular weight of 208.33 g/mol was obtained from Merck KGaA (Germany) with surfactant Cetyltrimethyl ammonium bromide (CTAB) C_19_H_42_Br obtained from the HAS HMRZEL laboratory (Netherland) loss drying 2% at 100°C as a liquid crystal templating. Ethyl alcohol absolute ((CH_3_)_2_CO) with molecular weight of 46.07 g/mol was purchased from ADWIC (EL Nasr pharmaceutical chemicals com, Egypt) and poly (vinyl alcohol) (PVA) (C_2_H_4_O)n with viscosity 25–32, degree of polymer 1700–1800 and pH 5–7 from LOHA SCHEME (India). Isopropanol CH_3_CH(OH)CH_3_, molecular weight 60.10 g/mol from ALPHA CHEMIKA (India).

### 2.2 Synthesis of silica nanoparticles

In this study silica nanoparticles were prepared using sol‐gel, micro‐emulsion and hydrothermal synthesis methods.

#### 2.2.1 Sol–gel method

A co‐solvent of 100 ml ethanol and 100 ml of distilled water (DW) was utilised for the hydrolysis of 20 ml TEOS. The solution pH was adjusted to ≈2 by droplets of HCl and then the sample placed in an oven for overnight at 70°C. The sample was afterword calcined at 600°C for 2 h. For further characterisation sample was grounded and kept in dissector.

#### 2.2.2 Micro‐emulsion process

In this method, two solutions (A and B) were prepared, firstly, solution A was obtained by dissolving of 4 g of PVA in of 250 ml DW and 150 ml ethanol during stirring at 70°C, and this was followed by addition of 0.8 g of CTAB to this solution and kept stirring till a transparent solution was obtained. Solution B, 25 ml TEOS added to 20 ml cyclohexane during stirring for 30 min. Furthermore, solution B was drop‐wised against solution A, during stirring for another 2 h in order to form the micro‐emulsion state. After centrifugation of the obtained mixture, the precipitate particles were dried at 70°C for overnight. Calcination process was conducted using the above mentioned conditions in sol–gel method.

#### 2.2.3 Hydrothermal synthesis

Solution of TEOS was prepared as 10 ml TEOS were dissolved in 80 ml DW with pH ≈ 2 by adding HCl and stirring for 2 h. The above solution was placed in an autoclave at 180°C for 24 h. After that, the sample was calcined at 600°C as mentioned above in sol–gel method. The samples utilised in this study were nominated according to Table 1.

**TABLE 1:** Samples codes utilised in this article

Sample	Preparation method
S1	Sol–gel
S2	Micro‐emulsion
S3	Hydrothermal

### 2.3 Materials characterisations

The microstructure, morphology and surface area characteristics of the formed samples were observed by Transmission Electron Microscope (TEM) and BET surface area analyser. Moreover, the physico‐chemical consistence was evaluated using XRD and FTIR analyses. In details, TEM, Hitachi HF‐2000, Tokyo, Japan with an accelerating voltage of 200 kV was employed to assess the morphology and diameter of the prepared MSNs samples. Particularly, few milligrams of sample was dispersed into co‐solvent of distilled water and ethanol (2:3). Copper grid was soaked in the previous suspension for very short time, which was allowed to dry in room temperature before the capturing of TEM images. The specific surface area and pore size distribution were acquired by automatic analyser (Quantachrome Nova Automated Gas Sorption System Version 1.12) by applying the method of Brunauer, Emmett and Teller (BET). Microscopic pore size distribution is performed by indirect molecular adsorption methods like nonlocal density functional theory (NLDFT) and N_2_ isotherms at 77 K. XRD assessments were obtained utilising a Diano X‐ray diffractometer using Cu Ka radiation (*λ* = 0.1542 Å) produced applying a tube voltage of 40 kV and a tube current of 40 mA (diameter 401 mm), accessorized with a Cu‐tube with excitation conditions of 36 mA and 45 kV, and a scintillation counter. FTIR curves were recorded in the range of 400–4000 cm^−1^ and at room temperature utilising Demonstrate 1600, Perkin‐Elmer USA. Practically, pellet composed of MSNs mixed with KBr pressed into in an evacuated die and the resulted pellet was examined by FTIR.

In addition, ZetasizerNano ZS, Malvern Instruments, UK accessorized with a 633 nm laser was utilised to assess the particle size and surface charges. Samples (50 mg) were suspended in 10 ml of deionised water; and were filtered using a 0.22 μm filter before analysis. A disposable capillary cell (DTS1060, Malvern) was loaded with sample prior to measurement process. Malvern instrument's dispersion technology software (Version 4.0) was used for data analysis and zeta‐potential values were estimated from the measured electrophoretic mobility data. The drug release performance of the different formed Nano silica was investigated in phosphate buffer saline (PBS; molarity 0.1 M and pH 7.4) that was detected by UV spectrophotometer at *ʎ* = 273 nm.

### 2.4 Drug loading and release behaviour

Silica nanoparticles (100 mg) was suspended in distilled water (100 ml) and stirred for a while and then 100 mg of doxycycline hyclate (dox) was dissolved in the silica suspension. To evaluate the in vitro drug release behaviour, 100 mg of dox‐loaded silica nanoparticles had been soaked in 100 ml of PBS buffer solution. To analyse the amount of drug release, at different time intervals 5 ml of solution were withdrawn up to 30 days. The same volume of fresh PBS was added to the soaked samples in order to maintain the sink conditions. The concentration of released dox was further determined by UV spectrophotometer at *ʎ* = 273 nm.

### 2.5 Cell viability

Cytotoxicity of tested samples was measured against Saos‐2 cells (a human osteosarcoma cell line, which possess many characteristics like osteoblastic cell such as producing mineralised matrix when they are injected under skin in animal moles) using the MTT cell viability assay. The extent of the reduction of MTT was quantified by measuring the absorbance at 570 nm [27]. In details, cells (0.5 × 105 cells/well), in serum‐free media, were placed in a flat bottom 96‐well microplate, and treated with 20 µl of serial concentrations of the tested samples (10–100 µg/ml) for 48 h at 37°C, in a humidified 5% CO_2_ atmosphere. At the end of incubation time, media removal was conducted followed by addition of 40 µl MTT solution (5 mg/ml of MTT in 0.9%NaCl)/well to the tested samples that was further incubated for additional 4 h. Acidified isopropanol (180 µl/well) was added to solubilise MTT crystals, which was followed by shacking of the plate at room temperature. Microplate ELISA reader was used afterward to determine the photometric absorbance at 570 nm. The average concentration of four repeats was calculated for each sample and the determined relative viability (%) was expressed in comparison with the normal cells. Cell viability was calculated according to Equation (1).

(1) $$\text{Percentage}\;\text{cytotoxicity}=\frac{1-av\left(x\right)\;}{\left[av\left(NC\right)\right]}\times 100$$

where, the average absorbance *av*(*x*) recorded at 595 correlated to wavelength of 690 nm; *NC* is the absorbance recorded for negative control at the same wavelengths.

## 3 RESULT AND DISCUSSION

### 3.1 Size and morphology of the MSNs

**FIGURE 1:**
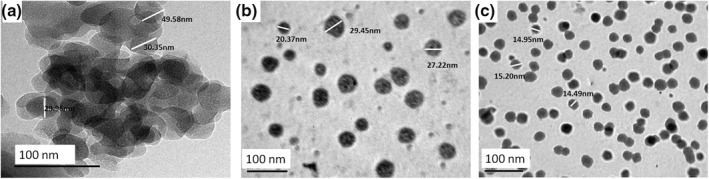
The effect of preparation method on the size and morphology of MSNs illustrated by TEM images of (a) S1, (b) S2 and (c) S3

The effect of preparation methods on the size and shape of MSNs was investigated by TEM micrographs, which are demonstrated in Figure 1. Based on these images, MSNs (samples S1) appear as semi‐irregular nano‐spheres in a form of agglomerations as observed at Figure 1a. The average diameter of S1 is about approximately 50 nm but for S2 and S3 it is about approximately 30–20 and 15 nm, respectively, in great dispersed manure as illustrated in Figure 1(b) and (c). Both samples S2 and S3 appear as spherical nanoparticles and characterised by low aggregation and well dispersion. This variation is thought to be related to the change of the nucleation and growth rates of the generated MSNs of each utilised method of preparation [28] as well as presences of polymer (micro‐emulsion method) that acts as a capping agent to prevent the particles agglomeration and to maintain their spherical shape.

The obvious changes in the particles size and shape are owing to the particle growth that takes place upon continued hydrolysis and condensation reaction caused by the concentration of hydroxyl group. In the Ref. [29] it is reported that, the amount of OH group was influenced by the volumetric ratio of ethanol to water. Thus, particle diameter and particle size distribution of silica powder were affected by regulating the quantity of ethanol and water involved in the preparation method. On the other hand, S2 prepared by micro‐emulsion, there are several parameters in this method that have an effect on the final size and morphology of silica powder during the preparation process. For example, some of these parameters are pH value of the aqueous phase, the viscosity of the external oil phase, type and concentration of the surfactant (cationic, ionic and nonionic surfactants), the diameter of micro‐emulsion droplets and (water to surfactant) molar ratio [30, 31, 32–30, 31, 32]. By adjusting these parameters, the number of nucleation sites and growth rate of the resulting silica nanoparticles could be controlled [33]. In relation to S3 prepared by hydrothermal method, silica nanoparticles were obtained under specific conditions including reaction temperature, pH value, solution concentration, ageing time and pressure [34, 35, 36–34, 35, 36]. According to a previous work [37], the reaction temperature and time possess great effects on the size of the produced particles along with the agglomeration between the synthesised particles. Therefore, decreasing the reaction temperature may lead to a decrease in the particle size as well as the agglomeration between MSNs, which explains the presence of particles with lower size.

**FIGURE 2:**
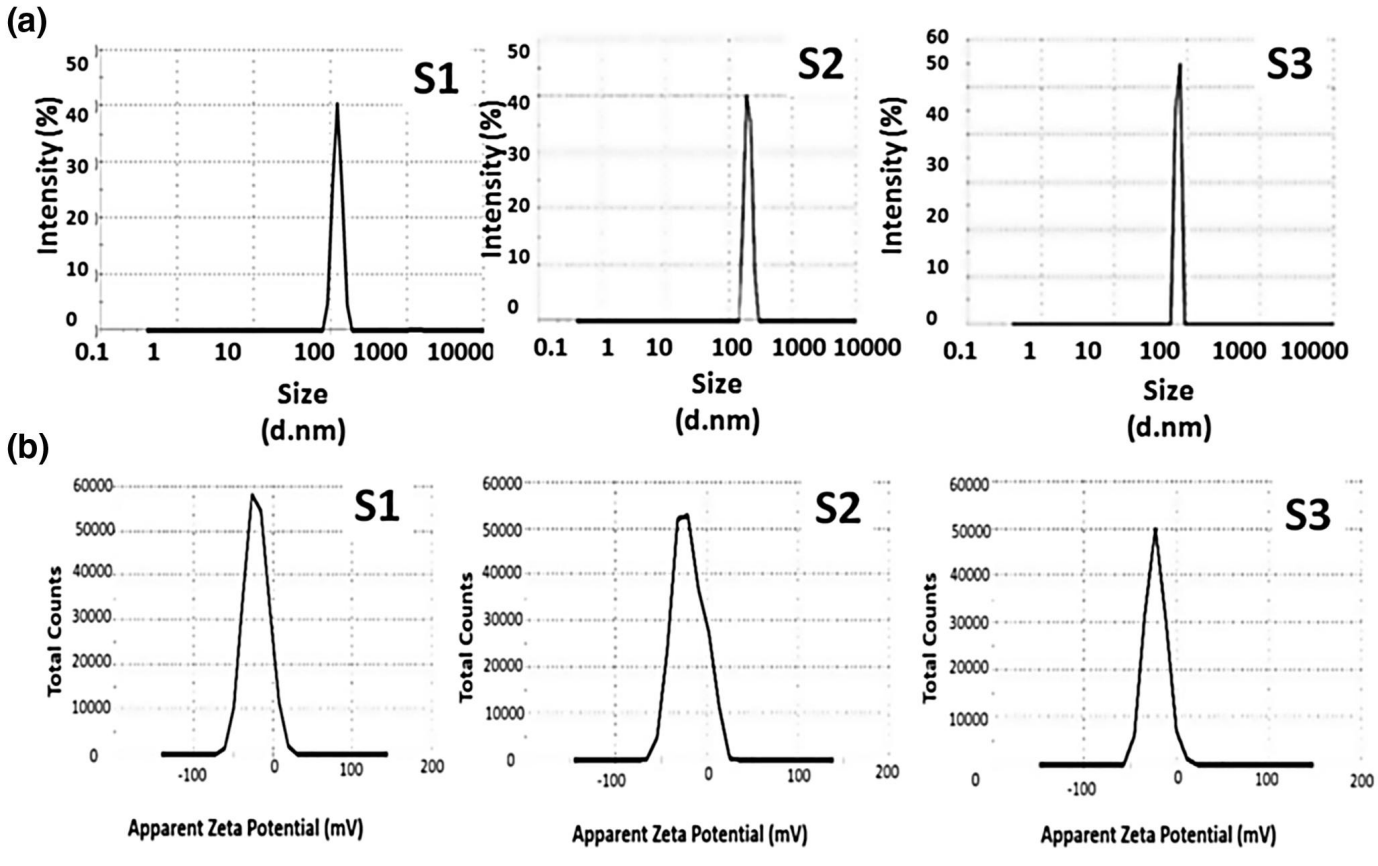
Represents the effect of preparation method on the (a) particles size distribution and (b) zeta potential of samples S1, S2 and S3

The measured diameters for the MSNs by TEM relatively contradicts with the data obtained from DLS, as we have, the average particle sizes are, respectively, 123.3, 191.3 and 72.2 nm for S1, S2 and S3 as shown in Figure 2a. This could be owed for the different techniques of sample preparation for both TEM and DLS along with the long‐time of suspension of nanoparticles while been measured by DLS, thus allows particles agglomerations to take place. Moreover, reaction time played the main role in the transformation of phase from hydroxides to oxides. From the obtained data, there is a noticeable influence on the particle size and morphology between prepared samples due to different preparation methods. In addition, zeta results confirmed the effect of the preparation method as could be observed from Figure 2b. It can be obviously noted that, the higher the specific surface area the higher change in zeta potential is obtained toward more negative values.

**FIGURE 3:**
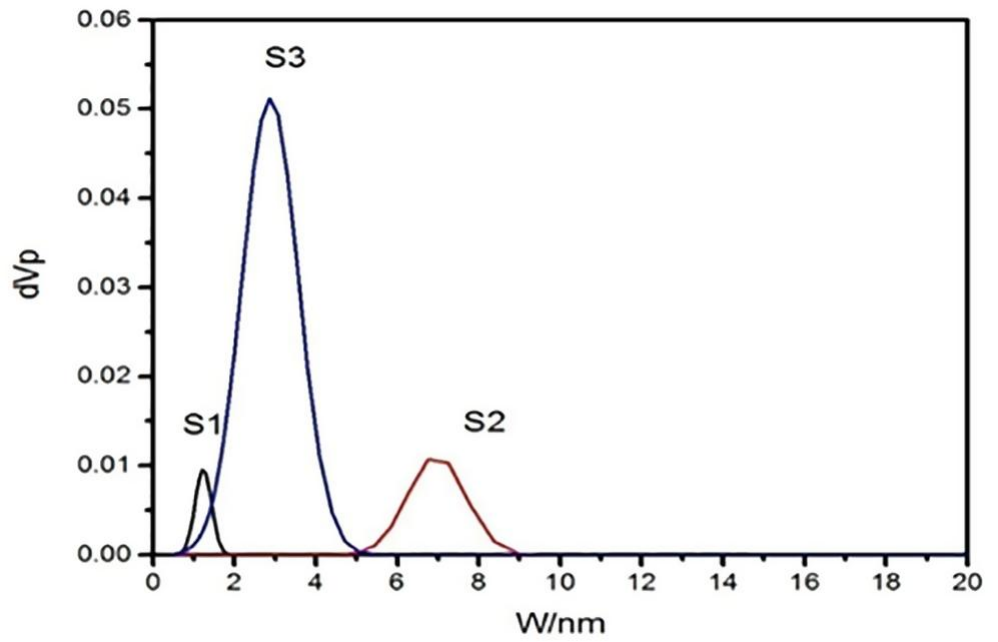
Demonstrates pore size distribution of MSNs computed using the BJH method

Furthermore, from the BET surface area measurements (Table 2), it can be noted that, sample S1 possess a variety of pore diameters, from 0.5 to 1.9 nm and surface area of about 111 m^2^/g. For pore diameter of sample S2 it is slightly extended from 4.5 to 9.5 nm with mean pore diameter approximately 9 nm and surface area of approximately 164 m^2^/g which relatively larger surface area when compared with S1. In contrast, the pore size distribution of sample S3 that was relatively smaller with range of 1–5 nm (Figure 3), S3 possess high surface area approximately 539 m^2^/g which considered higher surface area when compared with the other two samples (S1 and S2).

**TABLE 2:** Experimental data obtained from N_2_ adsorption–desorption isotherms

Sample	Mean pore diameter (nm)	Pore volume (cm^3^/g)	BET surface area (m^2^/g)
S1	1.831	0.051	111
S2	9.153	0.052	164
S3	3.578	0.481	539

**FIGURE 4:**
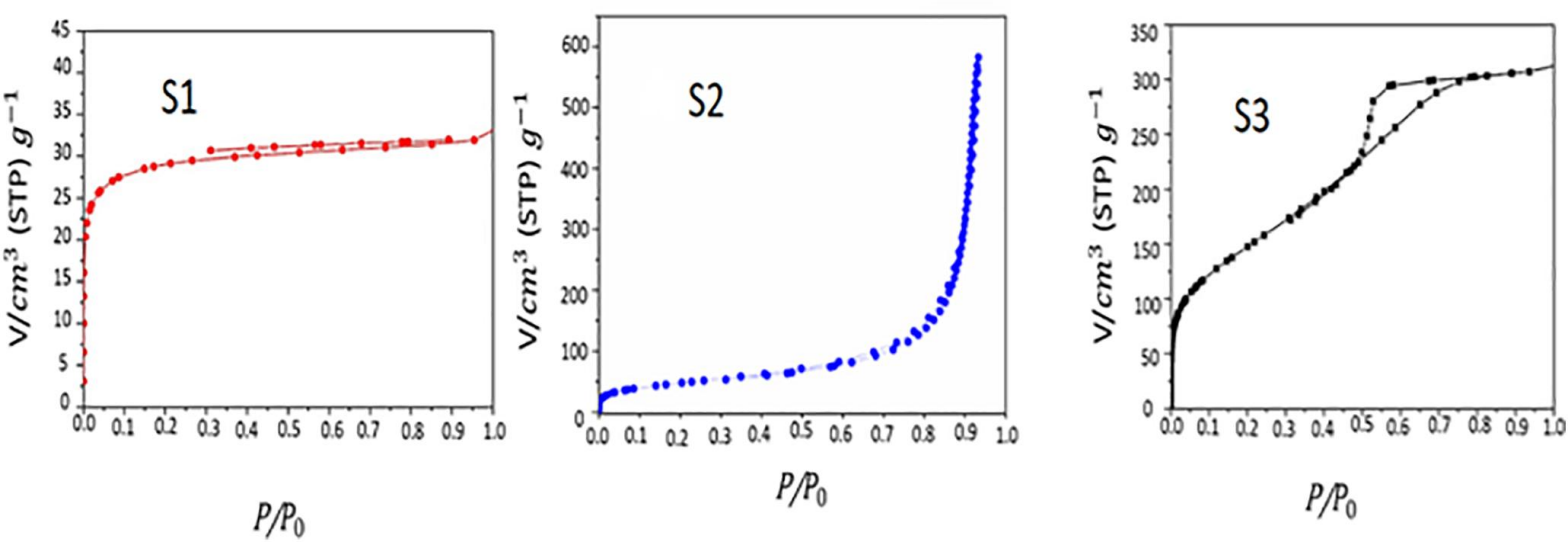
The effect of preparation method on the MSNs N_2_ adsorption/desorption isotherm analysis for the prepared samples

The surface area is one of the most important physical factors that should be considered along with particle size, pore volume and average pore diameter owing to their importance as evaluating parameters for success of the preparation method. Therefore, it is important to study the surface area of materials used in biological applications, especially in drug delivery applications. N_2_ adsorption/desorption isotherms were employed to identify three parameters related to pores, the type of pores whether open or closed, the pore diameter distribution and the form of pores, as shown in Figure 4. Based on IUPAC classification [38], the resulted isotherm curves for all samples exhibit hysteresis behaviour with two branches, adsorption (capillary condensation) and desorption (evaporation), indicates the presence of open pores between its particles. Sample S1 showed open hysteresis lope which indicate the formation of large irregular porous structured nanoparticles that allows fast and unclosed desorption as noted from Figure 4. The incomplete hysteresis loop of sample S1 is supposed to be a result of porous‐structure defect, which resulted in the loss of N_2_ adsorbed quantity right after the adsorption.

The hysteresis lope of sample S2 was matching with type II hysteresis loop as can be observed from Figure 4. At low relative pressures, the adsorbance sharply increased which indicating the presence of micropore structure. As the pressure increased, the monolayer adsorption translated to multilayer, and because of well‐developed macropore, the adsorbance sharply raised when the relative pressure value reached 1. Moreover, the maximum absorbance for adsorption and desorption branch of type H3 was not detected as this is commonly happened with open‐wedge pores.

On the other hand, the isotherm curve for S3 was matching to type IV hysteresis loop, thus indicating the formation of silica structure containing micropore and mesopore [39]. This is obvious in Figure 4. In addition, a broad distribution of pore diameter in S3 covering the range of micropore (<2 nm) and mesopore (>2 nm) was also observed, which is in consistent with results reported in the literature [40]. Typically, the pore shape of samples S3 is ascribed as the bottle‐neck pores that give rise to appear as a type H2 hysteresis loop according to IUPAC. This is explained by the presence of an inflection point followed by a sudden decline in the desorption branch.

### 3.2 Physicochemical properties

**FIGURE 5:**
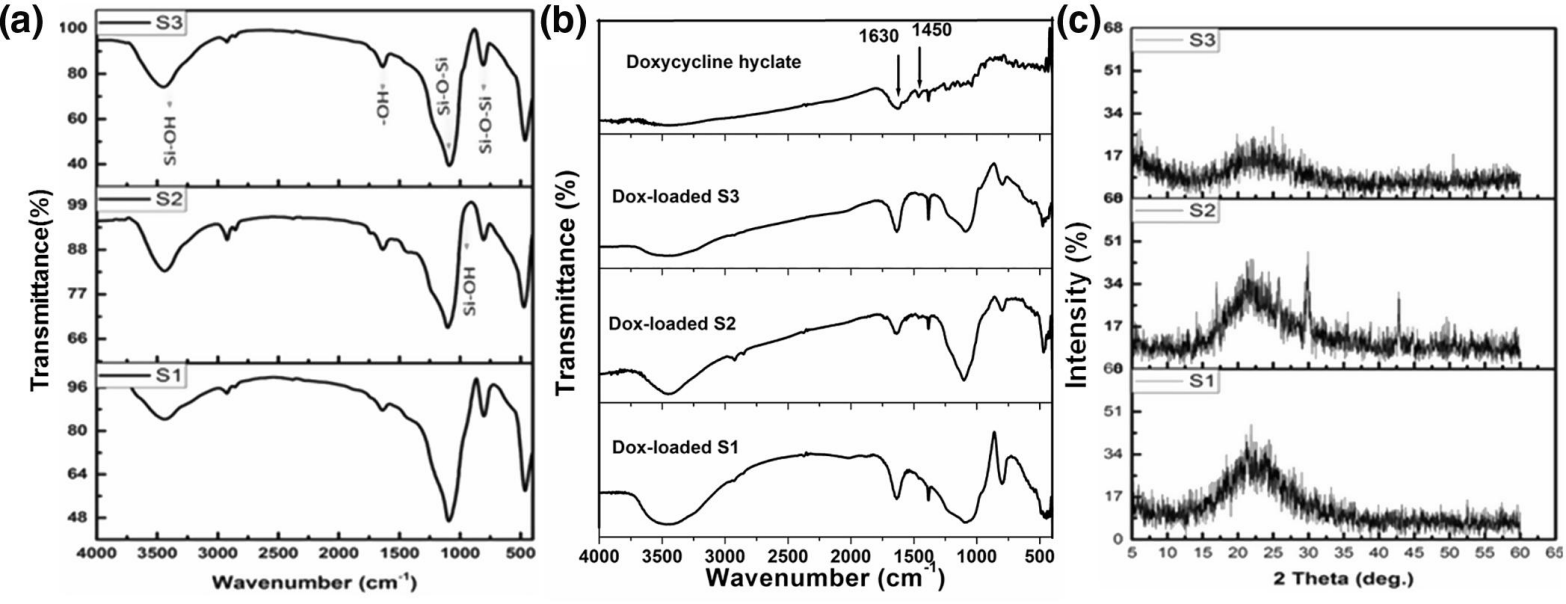
Represents FTIR spectra (a) before drug loading and (b) FTIR spectra of Dox‐loaded samples compared with native drug as well as (c) XRD patterns of samples S1, S2 and S3

FTIR analysis was applied to investigate the functional groups of the synthesised samples within the range of 400–4000 cm^−1^, as shown in Figure 5a. The dominant bands at 462, 832, 1091 and 1230 cm^−1^ are related to the bending and stretching vibrations of the silicon–oxygen bond, namely, the bending vibrations of Si–O at approximately 462 cm^−1^, the symmetric stretching vibrations of Si–O at approximately 802 cm^−1^, the asymmetric vibrations of Si–O–Si at approximately 1091 and approximately 1230 cm^−1^, these four bands were observed for all samples [41]. The weak absorption band at approximately 950 cm^−1^ corresponds to Si–OH bending vibration in S1 and S3. Small absorption band at approximately 1635.34 cm^−1^ assigned to O–H stretching vibrations of weakly bound water [42, 43]. The presence of OH groups could be referred to the water that was physically adsorbed on the sample from the air after calcination (see Table 3). For S2 sample additional bands were noted including CH_2_‐CH bond that was detected at 2800 cm^−1^ and less intense band at 1090–1150 cm^−1^ corresponds to HC–OH bond along with band observed at 800 cm^−1^ that is attributed to O–H bending mode. These bands suggest the existence of some PVA and CTAB residuals from the preparation process [24]. However, for both S1 and S3 samples, the detected bands are corresponding only to pure silica nanoparticles, which is consistent with the previous reported research work [44]. This suggested that, the preparation methods have no influence on the chemical integrity of the prepared silica nanoparticles. In order to confirm the Dox loading FTIR spectra were recorded for MSNs after drug loading and compared with the native drug as illustrated in Figure 5b. The Dox‐loaded samples have demonstrated two distinct bands at 1630 and 1421 cm^−1^ because of stretching of asymmetric –COO– and symmetric –COO– of the drug, respectively.

**TABLE 3:** FT‐IR functional groups observed for samples S1, S2 and S3

Functional groups	Absorption band range (cm^−1^)	Absorption band (cm^−1^)		
		S1	S2	S3
Bending vibration of Si–O–Si	457–476	462	472	462
Symmetric vibration of Si−O	798–800	802	804	806
Stretching vibration of Si−OH	950	950	950	950
Bending vibration of H−O–H	1632–1633	1639	1635	1637
CH_2_–CH	2800	–	2800	–
HC–OH	1090–1150	–	1090–1150	–

XRD curves of the different silica nano‐samples after heat treatment are shown in Figure 5c. It is worthy noted that, all the samples exhibit a wide amorphous hump in the range of 15°–30° with maximum intensity at 2*θ* = 23° (JCPDS card no. 45‐0130). However, sample S2 possess small diffraction peaks at 2*θ* = 30° and 43°, which might be due to the presence of some residual impurity from the surfactant or PVA polymer. This result is in good agreement with previous work [24], as they got some crystalline peaks in the XRD patterns of silica nanoparticles prepared in the presence of different amount of CTAB as a surfactant [24]. On the other side, the presence of the broad hump is referred to the existence of particles with small sizes and incomplete inner structure. As a result, we can say that, a large percentage of these particles are amorphous. Without any impurity peaks, pure samples of silica S1 and S3 nanoparticles were produced. It is worthy highlighting that the MSNs with amorphous curves are originated from surfactant free methods (in our study sol–gel and hydrothermal techniques). In addition, all methods produced the same amorphous hump within the same range, which implies the perfect mesoporous structure with few caves and pore‐diameter changes along with excellent thermodynamic stability [3].

This suggested that both sol–gel and hydrothermal synthesis methods have no remarkable effect on the physical consistence of the prepared silica nanoparticles. While very slight effect on the physical consistence of the silica nanoparticles produced by micro‐emulsion technique due to the presence of CTAB and PVA residuals.

### 3.3 Drug release behaviour

The amount of drug released into the PBS buffer solution was determined by UV spectrophotometer at *ʎ* = 273 nm.

**FIGURE 6:**
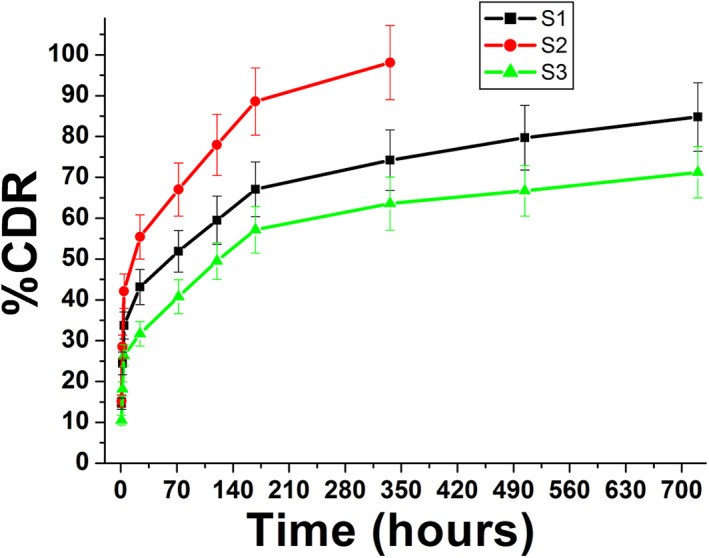
The effect of preparation method of MSNs on the drug release profiles S in PBS for different time intervals

The cumulative drug release (%CDR) profiles of doxycycline hyclate released from MSNs formulations are shown in Figure 6. From the release profiles it was revealed that, there is an obvious difference between release rates of all drug‐loaded samples. However, the release profiles of samples S1, S2 and S3 demonstrates prolonged burst release, respectively, of 10%–45% and 35% within 30 h, and it was linear up to 350 h for S2 and 720 h for S1 and S3. About 60% of the drug was released from sample S1 within approximately 170 h. While the same amount of drug was released from sample S2 only after approximately 40 h of submersion in PBS and most of drug was released within approximately 330 h. In addition, sample S3 showed slightly lower release rate than for sample S1 with 50% drug release in approximately 120 h.

Comparing the release behaviour of the prepared samples as a main factor of the current research, the ability of nanoparticles to control drug release, are clear to be adjusted by various parameters. The main factor in this work is nanoparticles surface characterisation specifically surface area and pore volume. Whereas mainly the sample S3 with highest pore volume and surface area and relatively sample S2 had showed good characteristics to be a good drug carrier for sustained treatments. On other side, sample S2 with medium surface area showed rapid release of drug and considerably highest drug release efficiency. This behaviour could be interpreted owing to the size of the pores is much bigger than the drug molecules, which means that most of the drug molecules will be adsorbed onto the surface of MSNs. This would cause the carried drug molecules to be released at relatively higher rates. Ideally, it is hypothesised that the sustained release of drug could be achieved when the carried drug molecules and pore diameter are approximately the same size. Similar results were early reported for the great influence of these parameters on the drug release profiles [45, 46, 47, 48–45, 46, 47, 48].

### 3.4 Drug release kinetics

The drug kinetics release for the drug‐loaded samples was fitted using different mathematical models and the kinetic parameters were recorded in Table 4. The best‐fit release kinetic data with the highest values of *R* ^2^ (regression coefficient) were shown by Korsmeyer–Peppas models. The values of *n* (diffusion exponent) were in the range of 0.234–0.276 (<0.5) indicating Fickian release (controlled diffusion) [49]. *R* ^2^ data indicate that diffusion model also suitably describe the release mechanism of model drug from sample S2.

**TABLE 4:** Release kinetics parameters of obtained by applying the selected mathematical models for the release data

Formula code	*R* ^2^‐value^†^			Korsmeyer‐Peppas model		*n*	k*	RE_0–720 h_^‡^ (%)
	Zero‐order	Diffusion	Korsmeyer–Peppas	t_50_** (hours)	t_90_*** (hours)			
S1	0.724	0.910	0.949	154.862	640.807	0.234	19.336	84.783
S2	0.747	0.918	0.919	48.746	233.529	0.276	21.307	98.102
S3	0.728	0.915	0.949	282.432	826.490	0.258	14.059	71.247

*Note. n* is the diffusion exponent, k* is the release rate constant, *t*_50_** is time required for 50% of the drug to be released, *t*_90_*** is time required for 90% drug release, ‡RE_0–720 h_ is the release efficiency of drug from 0 to 720 h and †*R* ^2^ value is the value for regression co‐efficient.

### 3.5 Cytotoxicity

**FIGURE 7:**
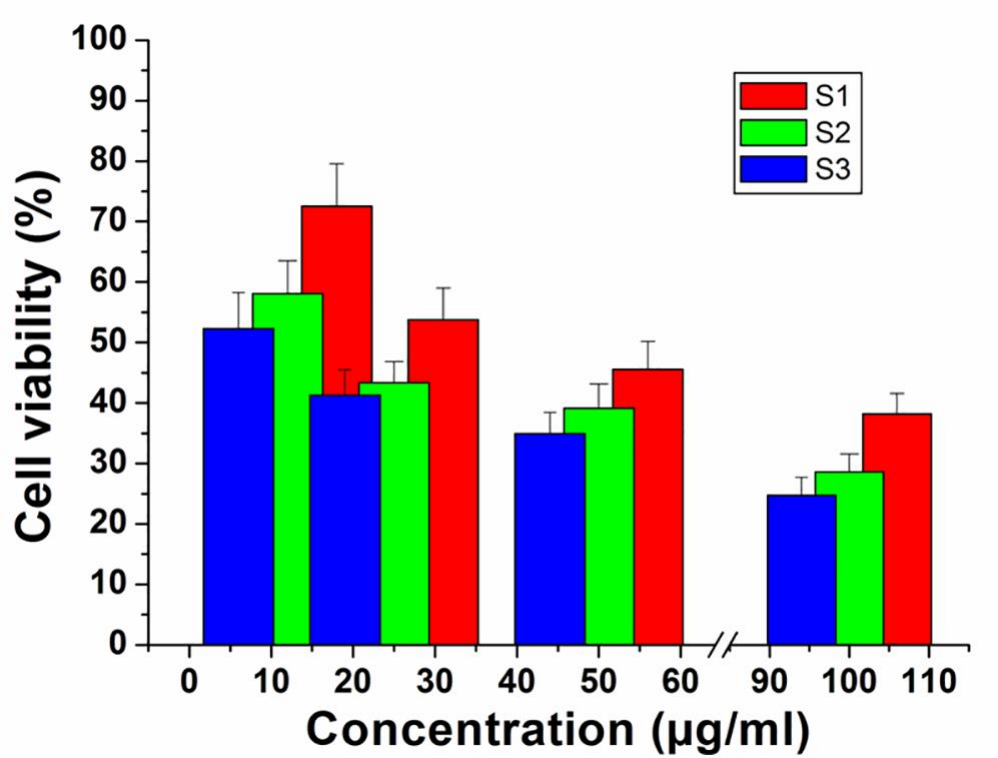
A comparative cytotoxicity study of S1, S2 and S3 against Saos‐2 determined for different concentrations of samples

Using MTT assay, the effect of the silica different samples on the growth of Saos‐2 cells was investigated after 72 h of incubation (*n* = 4). The obtained data were expressed as the mean value of cell viability (% of control). Saos‐2 cells exposed to S1, S2 and S3 for 72 h at dose levels of 12, 25, 50 and 100 µg/ml showed decreased cell viability as a function of concentration as shown in Figure 7. Incubating at lower concentration 12 µg/ml of sample S3, the cell viability was about 53.5%. Increasing in cell viability approximately reaches 78% after incubation of 12 µg/ml of samples S1 and S2, which means acceptable compatibility and safety for these samples. It is worth to highlight that the presence of residual impurities in sample S2 has decreased the cell viability to 58.3%, which consider lower than S1 but still higher than S3. This implies that they have great potentiality to be used as a drug carrier than sample S3. This result suggested the great influence of the preparation method on the materials–cell interactions.

## 4 CONCLUSION

Preparing amorphous and semi‐crystalline silica nanoparticles for drug carrier purpose was achieved in the current study by sol–gel, micro‐emulsion and hydrothermal synthesis techniques using the same precursor (TEOS).

Based on the obtained characteristics for each silica nanoparticles numerous therapeutic applications could be explored. Hydrothermal technique produces MSNs with very small particle sizes (≈15 nm) with the highest surface area (538.72 m^2^/g), while the bigger particle sizes (≈50 nm) and the smaller surface area (111 m^2^/g) were recorded for sol–gel technique. This recommends the superiority of hydrothermal technique over the sol–gel, especially if the neurological disorders are targeted as they require carrier particle sizes greater than 20 nm. The changing of the properties of the samples prepared depends greatly on the method of preparation as was confirmed by the surface area, size and biocompatibility of the MSN nanoparticles. The medication delivery results indicated quicker delivery from MSNs (micro‐emulsion) that contrasted with the other two samples (sol–gel and hydrothermal techniques), which make it more appropriate in cases which require huge portions for brief periods. On the other side, MSNs nanoparticles prepared by hydrothermal technique are recommended for long time medication delivery. Based on the desired application the method of preparation of MSNs could be selected as determined in the current research.
